# Supplementary material for: The interactome of intact mitochondria by cross-linking mass spectrometry provides evidence for coexisting respiratory supercomplexes
Source: Mol Cell Proteomics. 2017 Dec 8;17(2):216–32. doi: 10.1074/mcp.RA117.000470 (PMC5795388; doi:10.1074/mcp.RA117.000470)
Supplement: Supplemental Data [file supp_RA117.000470_133922_0_supp_23978_fzffwf.zip › spectra_annotation/mito_DR_spectra_annotation/172-1-10-1-5-1.pdf]

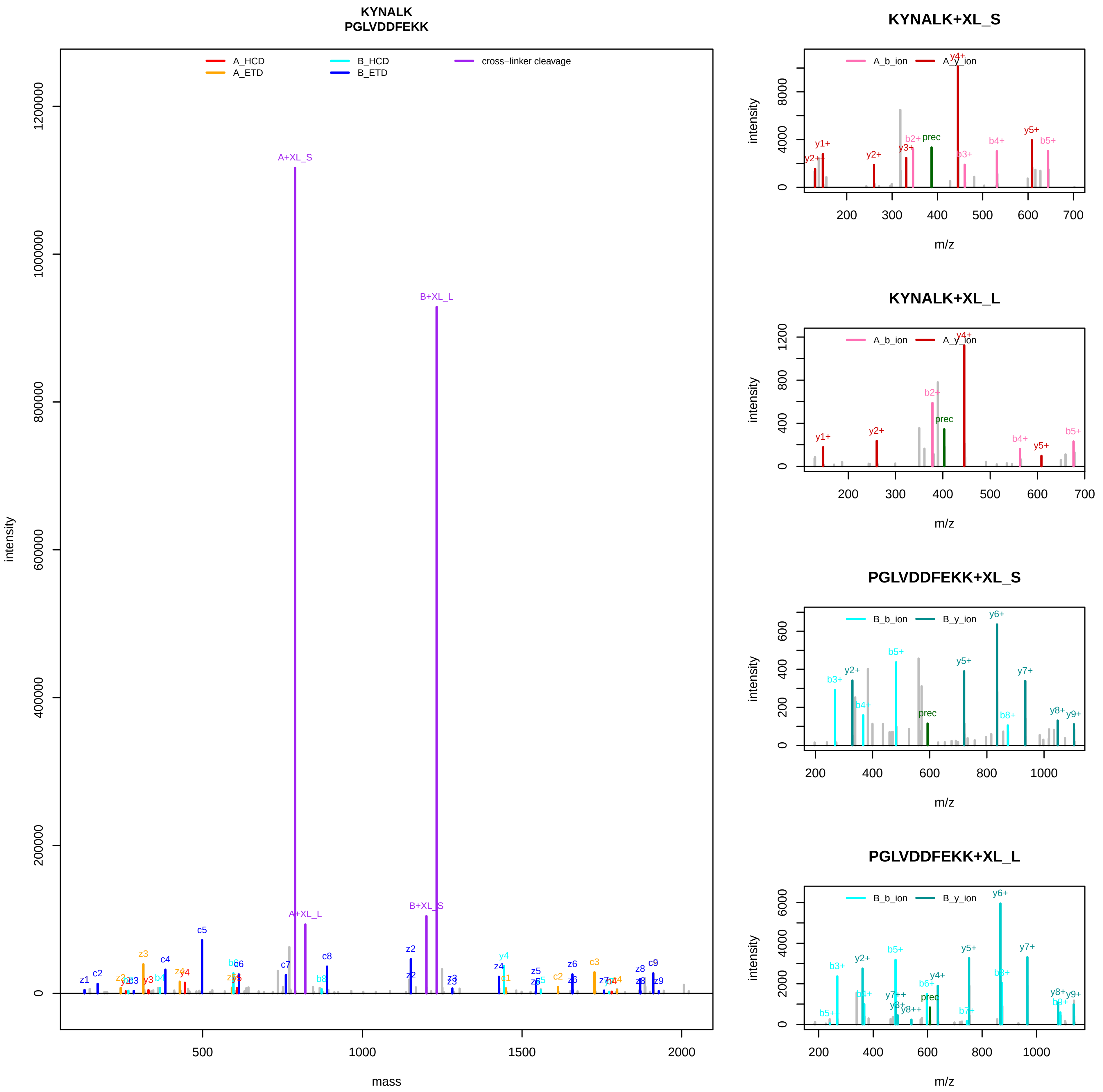
KYNALK
PGLVDDFEKK
A_HCD
A_ETD
B_HCD
B_ETD
cross−linker cleavage
intensity
mass
0
200000
400000
600000
800000
1000000
1200000
500
1000
1500
2000
A+XL_S
B+XL_L
A+XL_L
B+XL_S
c5
c4
c2
z1
z3
c7
c8
z2
c9
z8
z9
y4
z4
z5
z6
c3
c2
c1
c6
y4
KYNALK+XL_S
A_b_ion
A_y_ion
y4+
y1+
y2+
y2++
y3+
b2+
prec
b3+
b4+
y5+
b5+
KYNALK+XL_L
PGLVDDFEKK+XL_S
B_b_ion
B_y_ion
y6+
y7+
y8+
y9+
b8+
PGLVDDFEKK+XL_L
m/z
200
300
400
500
600
700
800
1000
